# Evaluation of serological test of Zika in an endemic area of flavivirus in the Colombian Caribbean

**DOI:** 10.1186/s12941-019-0328-7

**Published:** 2019-10-14

**Authors:** German Arrieta, Salim Mattar, Yeneiris Villero-Wolf, Luty Gomezcaceres, Amanda Doria

**Affiliations:** 1 Instituto de Investigaciones Biológicas del Trópico, Universidad de Córdoba, Montería, Córdoba, Colombia; 2 Clínica Salud Social, Sincelejo, Sucre, Colombia; 3 Grupo de Salud Pública, Corporación Universitaria del Caribe-CECAR, Sincelejo, Sucre, Colombia

**Keywords:** Diagnosis differential, Reagent kits, Diagnostic, Epidemiology, Arbovirus infections, Tropical medicine

## Abstract

**Abstract:**

The Zika virus (ZIKV) is an emerging flavivirus transmitted primarily through arthropods, endemic in Africa, Asia, and the Americas, and is considered a global threat by the World Health Organization.

**Objective:**

To evaluate a commercial Zika virus test (IgG/IgM catalog number B815C, Biocan, Canada.

**Methods:**

We evaluated 30 sera of patients diagnosed with Dengue, Leptospira, Malaria, Hantavirus, and Chikungunya. To establish the sensitivity of the test, two groups of sera were analyzed, the first one was patients with Zika RT-qPCR positive, and the second were patients RT-qPCR negative but with clinical suspicion of Zika.

**Results:**

The specificity was of 23.3% (7/30), the sensitivity in acute patients with positive RT-qPCR was of 63.6%, the patients with clinical suspicion of Zika the sensitivity (IgM) was of 80% (n = 8/10). Overall sensitivity (IgM) of both groups was of 71.4% (15/21).

**Conclusions:**

The test showed a low specificity to be used as a serological test in an endemic area of flavivirus infection.

## Introduction

The Zika virus (ZIKV) is an emerging flavivirus transmitted primarily through arthropods, endemic in Africa, Asia, and the Americas, and is considered a global threat by the World Health Organization [1]. It has been shown that ZIKV infection during pregnancy can present neurological complications, microcephaly, intracranial calcifications, and ocular abnormalities in the fetus [2].

In the Americas, Dengue (DENV), Chikungunya, ZIKV, and other encephalitis viruses are mosquitoes borne diseases that circulate in tropical countries [3, 4]. Currently, in Latin America using only clinical criteria, DENV, ZIKV, and Chikungunya are improbable to make a definitive diagnosis and distinguish from other infections that cause similar systemic febrile illness [5, 6]. The similarity in clinical appearances and the potential for life-threatening fetal outcomes, including microcephaly, congenital neurologic malformations, and fetal decease, and other, neurological manifestations such as Guillain–Barré syndrome (GBS), emphasize the importance of accurate ZIKV diagnostics [5, 7, 8].

ZIKV was introduced in Colombia in September 2015, causing severe public health problems that included the appearance of cases of microcephaly in newborns to mothers who had the infection during pregnancy as well as the occurrence of cases of GBS in adults and children with acute infection of ZIKV [7, 9]. Currently, Colombia has reported 108,948 cases of ZIKV, 342 cases of children with microcephaly associated with ZIKV infection and 461 cases of GBS with a history of ZIKV infection [10, 11]. Approximately 9% have been diagnosed with laboratory molecular tests [11]. Additionally, co-infections are common in the tropics, and the clinical manifestations, and even histopathological findings are comparable and challenging to make a differential diagnosis [6].

Currently, the diagnosis of ZIKV is carried out with direct methods to detect viral RNA, such as RT-qPCR and viral isolation, which are considered as the gold standard for the definitive diagnosis. Viral RNA detection should be performed in serum or plasma within 10 days of the onset of the disease, in whole blood within 3 weeks of onset and semen up to 3 months [12].

Real-time reverse transcription-polymerase chain reaction (RT-qPCR) is the method more used by diagnostic laboratories because of high sensitivity and reproducibly detecting targets as low as 10 copies of extracted RNA [13]. However, RT-qPCR requires trained staff and high-cost reagents. A rapid, sensitive, specific, and accessible diagnostic test for the detection of IgM and IgG antibodies to ZIKV is important in developing countries affected by arboviruses. Nevertheless, the immunologic cross-reactions of ZIKV in endemic areas with the presence of other flaviviruses represent a problem for this type of tests, because most patients than presenting clinical suspicion of ZIKV are misdiagnosed; this is due to the extensive similarity of amino acids of these flaviviruses [14].

Among the rapid tests available, there is a lateral flow chromatographic immunoassay (Biocan Tell Me Fast Zika Virus IgG/IgM Rapid Test), the test cassette consists of a pink-colored conjugate pad containing recombinant (Zika NS1 protein and envelope protein) common antigens conjugated with colloid gold and rabbit IgG-gold conjugates. A nitrocellulose membrane strip containing two test bands (T1 and T2 bands) and a control band (C). The T1 and T2 bands are pre-coated with monoclonal anti-human IgM and IgG, and the C band is pre-coated with goat anti-rabbit IgG.

This study aimed to evaluate the sensitivity and specificity of the commercially available test for the rapid detection of IgM and IgG antibodies against ZIKV in the Colombian Caribbean.

## Methods

Zika Virus IgG/IgM Antibody Rapid Test, (Catalog number B815CO72916, Biocan, Canada) was assessed using the manufacturer’s instructions; 25 µl of patient’s serum and two drops of the buffer of the kit were used, 20 min later results were read. To establish the sensitivity of the test, 21 sera of patients in the acute phase (1–7 days) with clinical symptoms compatible with ZIKV were used (Table 1, groups^a,b^). Of these 21 sera, 11 were ZIKV positive by RT-qPCR and 10 ZIKV negatives by RT-qPCRb (Table 1, groups^a,b^). Sera were collected between 2015 and 2016, and the specimens belonged to patients who were seen at the Clinica Salud Social of Sincelejo, Colombia, and the Biological Research Institute of the Tropic of the University of Córdoba, Colombia. As inclusion criteria patients with exanthema and at least one of the following signs or symptoms: fever, arthralgia, myalgia, and conjunctivitis were included [15, 16]. Additionally, 12 sera of patients in convalescence stage were evaluated for the presence of IgG and IgM antibodies (Table 1, group^c,d^). Of these 12 sera, 6 patients had RNA ZIKV by RT-qPCR (range 4–11 months), and 6 patients had a high clinical suspicion of ZIKV, but negative by RT-qPCR (range 3 weeks–7 months). To establish the specificity of the test, 30 sera in acute stage collected between 2012 and 2014 (no ZIKV circulation in the Americas), diagnosed as Dengue (n = 19), Leptospira (n = 3), Malaria (n = 3), Hantavirus (n = 3) and Chikungunya (n = 3) were analyzed; Differential routine diagnostic tests to Dengue, Leptospira, Hantavirus, Chikungunya, and Malaria thick smear were performed (Table 1).

**Table 1:** Evaluation of ZIKV virus test IgG/IgM

Patients groups	Samples (year collection)	Positive results of ZIKV test		
		n	IgM	IgG
Tropical fever groups with no ZIKV circulation n = 30	Dengue (2012)	19	18	2
	Leptospira (2012)	2	1	1
	Malaria (2012)	3	1	1
	Hantavirus (2012)	3	2	1
	Chikungunya (2014)	3	1	0
	Total	30	23	5
Epidemic ZIKV group with ZIKV circulation n = 33	ZIKV PCR positive^a^ (2015–2016)	11	7	0
	ZIKV PCR negative with high clinical suspicious^b^ (2015–2016)	10	8	0
	ZIKV convalescent non-acute stage^c^ (2015–2016)	6	5	0
	High clinical suspicious patients convalescent stage^d^ (2015–2016)	6	5	0
	Total	33	25	0

^a+b^ Sera were analyzed using the qRT-PCR kit (Zika virus polyprotein gene, genesig^®^ Advanced kit, Primer design Ltd., UK), viral loads were between 0.155–195 virus/μl

^b^ZIKV PCR negative with high clinical suspicious of ZIKV

^c^ZIKV convalescent non-acute stage confirmed by PCR (range 4–11 months)

^d^High suspicious clinical ZIKV patients in convalescent stage, no confirmed by PCR (range 3 weeks–7 months)

Regarding the molecular detection of ZIKV in serum, viral RNA was extracted from 140 μl of serum employing the QIAamp^®^ Viral RNA Mini kit (QIAGEN, Hilden, Germany) following the manufacturer’s instructions. In order to concentrate the virus RNA in the serum, we used Lenti-X^®^ concentrator (Cat 631231, Clontech). The RT-qPCR was performed with the kit Zika Virus polyprotein gene, genesig^®^ Advanced kit, (Primerdesign Ltd., United Kingdom). For each reaction, 10 µl of precision PLUS OneStep 2× RT-qPCR Master Mix were added; 1 µl of endogenous control primer/probe mix; 4 µl of RNase/DNase free water and 5 μl of RNA template. A standard curve to quantify the viral load was carried out, 5 μl RNase/DNase free water was used as a negative control. The amplification was performed using the CFX96 Touch Real-Time PCR Detection System (Bio-Rad). A serum specimen with a threshold cycle (CT) number ≤ 40 was considered to be positive for ZIKV.

## Results

The sensitivity of the IgM del test in patients with acute stage and positive RT-qPCR was of 63.6% (n = 7/11; IC 95% = 31–81%); in patients with high clinical suspicious of ZIKV but negative qRT-PCR, the sensitivity was of 80% (n = 8/10; IC 95% = 44–97%). The sensitivity de IgM of both groups was of 71.4% (n = 15/21; IC 95% = 48–89%) (Table 1, groups^a+b^). The specificity of the test was of 23.3% (7/30); the positive predictive value and the negative predictive value was 53.8% and 36.6% respectively. IgM antibodies were detected in 90% of patients between 1 and 2 days’ onset symptoms. In contrast, patients between 3 and 7 days’ onset symptoms, IgM was of 50% (5/10) (Table 2). Therefore, there was no correlation between days of clinical evolution and detection of IgM antibodies. The detection of IgM in patients in convalescent-phase (3 weeks–11 months, Table 1) was of 91.6% % (11/12). IgG detection was not observable in any of the samples tested, at any stage of infection (1 day–11 months) demonstrating a null sensitivity to IgG detection. There was no correlation and dependence between viral load and detection of IgM, in patients with high (195 viruses/ml loads) or low (0.151 viruses/ml loads) (Table 2).

**Table 2:** Days symptoms onset of Zika patients, viral load and serology test

Code	Days symptoms onset to sampling	ZIKV qRT-PCR (virus/µl)	Test result	
			IgM	IgG
Am 1	1	195	Positive	Negative
Am 3	1	0.155	Positive	Negative
Am 13	1	3.12	Positive	Negative
Am 2	1	0	Positive	Negative
Am 4	1	0	Positive	Negative
Am 6	1	0	Negative	Negative
Am 8	1	0	Positive	Negative
Am 9	1	0	Positive	Negative
U 9	2	0.83	Positive	Negative
U 13	2	0.23	Positive	Negative
Am 11	2	0	Positive	Negative
Am 12	3	6.04	Negative	Negative
Am 23	3	0.19	Positive	Negative
Am 27	3	1.05	Negative	Negative
U 11	3	0.31	Negative	Negative
Am 15	3	0	Negative	Negative
Am 14	4	13.7	Negative	Negative
Am 16	4	0	Positive	Negative
Am 5	6	1.29	Positive	Negative
Am 10	6	0	Positive	Negative
Coro 20	7	0	Positive	Negative

Diagnosis of ZIKV infection is carried out through the detection of viral components like RNA, proteins, virus isolation, and detection of antibodies. RT-qPCR is the most common assay because of its good sensitivity and specificity [17]. However, RT-qPCR in Colombia only is available in specialized laboratories. Therefore, the majority of cases are misdiagnosed or diagnosed as probable. Currently, in endemic countries mosquito’s vector-borne diseases like Colombia, a feasible and serologic accuracy test to make a reliable differential diagnosis from DENV, Yellow Fever and ZIKV are decisive to implement the public health programs.

## Discussion

The sensitivity of 71.4% of the present work appears to be satisfactory, especially in the acute stage of ZIKV infection. In a recent work of Zhang et al. [5], found IgG/IgA antibodies in early acute-phase ZIKV infections (< 6 days after symptom’s onset) in Dengue-endemic regions, their assay recognized 47% of ZIKV infections with high specificity [5]. Jeong et al. [18], performed the kits Euroimmun^®^ ELISA and Zika virus IgM/IgG Ab Rapid Test (Biocan), they found that kit Euroimmun^®^ ELISA was able to detect the IgM from day 2 until 41 days after symptom’s onset, the IgG was detected from day 8 until 52 days. In contrast, using the test IgM/IgG (Biocan) antibody detection was unsuccessful [18]. Contrary, in the present work using the test Biocan, we were able to detect IgM antibodies from the 1st day until 11 months. The IgG was not observable in any of the samples tested, demonstrating that the test cannot be performed in seroprevalence studies. It has not been established with precision the time of permanence of the antibodies in the serum of the patients infected by ZIKV, thus is an important include greater number of patients with past infections of ZIKV [19].

Recently, a multiplexed assay using nanotechnology gold platform for detecting IgG and IgA antibodies and IgG avidity against both ZIKV and DENV infections was developed [5]. Sera’s patient from endemic flaviviruses countries of Colombia and the Dominic Republic were analyzed, the assay demonstrated IgG and IgA antibodies against ZIKV non-structural protein 1 (NS1) antigen were specific to ZIKV infection, and IgG avidity shown acute ZIKV infection and past DENV-2 infection [5]. This kind of test would be valued in endemic regions.

A specificity of 23.3% for IgM demonstrates a cross-reaction of ZIKV with other flaviviruses like Dengue. We found that 94.7% (n = 18/19) of patients with DENV were positive for Zika Biocan IgM test. The 11 analyzed samples with diagnosed Malaria, Hantavirus, Leptospirosis, and Chikungunya were also false ZIKV IgM positives, indicating previous infections with DENV because IgG antibodies were observed. Hence, the presence of DENV or Yellow Fever antibodies in a primary infection may alter the specificity of the test. Similarly, we anticipate that cross-reactivity with antibodies generated by the Yellow Fever vaccine or a future Dengue vaccine may show potential IgG positive signal for a future non-specific ZIKV test. The design of such test addresses the issues of the prior presence or immunological memory for several other flaviviruses in the Americas. As Yellow Fever vaccinations are part of the Public Health program in Colombia, this study alerts the medical and scientific community to conduct careful testing and controls when new diagnostics tests will be introduced in the country. The low specificity of the test can be explained because Zika Biocan rapid test, as well as others commercially available kits, using a mixture of antigens NS1 (nonstructural protein 1) and ZIKV E (glycoprotein). ZIKV E has been shown in previous studies to have low specificity because it presents a high degree of similarity with the four serotypes of DENV [17]. Paul et al. [14] investigated the role of preexisting antibodies against DENV during ZIKV infection by analyzing the epitopes of human anti-DENV monoclonal antibodies (MAbs) and by using an Elisa capture tested for their ability to recognize the protein of surface ZIKV E glycosylated. The results showed that the anti-DENV HMAbs strongly recognize the surface glycoprotein ZIKV E, further demonstrating by immunostaining that these HMAbs also recognize cells infected with ZIKV [14]. In contrast, in other studies that have evaluated tests using antigen mixtures with NS1, better specificity has been observed, suggesting that the non-specificity of the test is not due to NS1 use, but to the antigen ZIKV E [19–21].

## Conclusion

The kit Zika Virus IgG/IgM Antibody Rapid Test needs to improve the specificity. If this assay is improved could facilitate a differential specific diagnosis of ZIKV infection and other flaviviral infections like Dengue, West Nile virus, Sant Louis encephalitis virus, and even Yellow Fever infections. The sensitivity assay could still have diagnostic value in the early acute phase (1–7 days). However, the test only can be used if it excluded the IgM active infection of Dengue in suspicious patients with ZIKV infection.

## Data Availability

No applicable.
